# Association Between Informal Caregiving and Changes in Cardiovascular-related Health Behaviors Among Middle-aged and Older Adults in Japan: A 15-year Panel Survey

**DOI:** 10.2188/jea.JE20240197

**Published:** 2025-08-05

**Authors:** Yuta Taniguchi, Atsushi Miyawaki, Masao Iwagami, Takehiro Sugiyama, Taeko Watanabe, Tomoko Ito, Nanako Tamiya

**Affiliations:** 1 Department of Health Services Research, Institute of Medicine, University of Tsukuba, Ibaraki, Japan; 2 Institute for Global Health Policy Research, Bureau of International Health Cooperation, National Center for Global Health and Medicine, Tokyo, Japan; 3 Department of Public Health, Graduate School of Medicine, The University of Tokyo, Tokyo, Japan; 4 Department of Health Services Research, Graduate School of Medicine, The University of Tokyo, Tokyo, Japan; 5 Health Services Research and Development Center, University of Tsukuba, Ibaraki, Japan; 6 Diabetes and Metabolism Information Center, Research Institute, National Center for Global Health and Medicine, Tokyo, Japan; 7 Department of Public Health and Nursing, Institute of Medicine, University of Tsukuba, Ibaraki, Japan

**Keywords:** informal caregiving, caregivers, health-related behaviors, Japan

## Abstract

**Background:**

Studies have shown that informal caregiving is associated with an increased risk of cardiovascular diseases. However, there is limited evidence on the mechanisms involved. To fill this knowledge gap, we investigated the association of informal caregiving with changes in health-related behaviors.

**Methods:**

We analyzed a nationally representative sample aged 50–59 years as of 2005 using fifteen waves of the Longitudinal Survey of Middle-Aged and Older Adults, which was conducted between 2005–2019. We investigated the association between the change in informal caregiving status and the change in health-related behaviors, including (1) heavy drinking, (2) smoking, (3) no exercise habits, and (4) no attendance at annual health checkups. We used multivariable logistic regression models with correlated random effects, adjusting for individual-level time-invariant characteristics.

**Results:**

Among 268,165 observations from 30,530 participants (median age 55; interquartile range, 52–57 years at baseline; 51.6% women), 32,164 (12.0%) observations from 10,224 individuals provided informal care. After adjusting for potential confounders, informal caregiving was associated with higher probabilities of deteriorating health-related behaviors, including heavy drinking (adjusted odds ratio [aOR] 1.16; 95% confidence interval [CI], 1.03–1.32; adjusted *P* = 0.032) and no exercise habits (aOR 1.09; 95% CI, 1.04–1.15; adjusted *P* < 0.001). We observed similar patterns for smoking (aOR 1.12; 95% CI, 1.001–1.26; adjusted *P* = 0.053) and no attendance at health checkups (aOR 1.05; 95% CI, 0.999–1.10; adjusted *P* = 0.053).

**Conclusion:**

This study showed that the transition into informal caregiving was associated with deteriorating cardiovascular-related health behaviors in Japan. These findings highlighted the importance of continued efforts to prevent the deterioration of caregivers’ health-related behaviors.

## INTRODUCTION

Informal caregivers—persons who provide care to their family members—play a major role in caring for people with long-term care needs. In Japan, 6.5 million (5% of the population) were providing informal care in 2021^1^ and 21% of informal caregivers provided care almost all day.^2^ Health-related problems among informal caregivers can diminish the well-being of caregivers, hinder continued caregiving, and lead to unemployment, particularly among the working-age population.^3^^,^^4^ As the population ages,^5^ the need for informal caregivers is expected to increase, leading to a growing health policy concern regarding the health status of caregivers. Studies have shown that informal caregivers are more likely to suffer from cardiovascular disease compared with non-caregivers.^6^^–^^9^ This is specifically evident among those providing care for long hours,^6^^,^^7^ those with higher levels of depression,^8^ and single men.^9^ Although the psychological distress involved with caregiving may lead to physiological responses and illness, such as increased stress hormones and elevated blood pressure, another potential explanation is that the change in behaviors may increase the risk of cardiovascular diseases.^10^

Mixed results have been reported on the association between informal caregiving and health-related behaviors.^11^ Some studies showed deteriorating health-related behaviors among caregivers, such as drinking,^12^ smoking,^13^^–^^15^ and decreased physical activities.^16^^–^^20^ However, others observed the opposite results, including less smoking,^12^ more physical exercise among caregivers,^13^^,^^14^^,^^21^ or no differences.^22^^,^^23^ A cross-sectional study from Japan reported lower participation in health checkups among those caring for a family member with a higher care-need level,^24^ while another study from the United States found no differences in the use of preventive services.^25^ While informative, most of these studies used a cross-sectional design and, therefore, were limited by confounding bias owing to the possibility that informal caregivers and non-caregivers may have different preferences and attitudes toward health in unmeasurable ways. Furthermore, previous studies did not evaluate how the association varied by caregiving intensity. Understanding the association between informal caregiving and health-related behaviors may lead to interventions that effectively mitigate the health effects of caregiving. In this context, we sought to answer the following three key questions using longitudinal panel data of nationally representative middle to older age samples in Japan. First, is the change in informal caregiving status within individuals associated with health-related behaviors, such as heavy drinking, smoking, limited exercise habits, and no attendance at annual health checkups? Second, do these associations vary by the intensity of caregiving? Third, do these associations vary by gender and socioeconomic status, such as educational attainments and income level?

## METHODS

### Data source

We used the 15-year panel data for 2005–2019 extracted from the Longitudinal Survey of Middle-Aged and Elderly Persons, a Japanese nationally representative population-based survey conducted by the Ministry of Health, Labour and Welfare (MHLW).^26^ The survey has collected information on family situation, health conditions, employment status, and economic conditions every year since 2005. In the first wave, the MHLW recruited all individuals aged 50–59 as of October 2005 in the randomly sampled 2,515 districts throughout Japan (40,877 individuals). The MHLW distributed the questionnaire directly (up to the fifth wave) or by mail, and the individuals who consented to participate in the survey filled out and returned the questionnaires. The response rate of the first wave was 83.8% (34,240/40,877), and the second to fifteenth waves were conducted on the respondents who took part at least once in the previous two waves. As of the fifteenth survey in 2019, the follow-up rate was 48.8% (19,931/40,877).

### Study population

In this study, we included individuals who participated in the survey for at least two consecutive waves to obtain information on the exposure and outcome variables. We excluded participants whose exposure, outcome, or covariate variables were missing (11.5%). This study was approved by the Ethics Committee of the University of Tsukuba (approval no. 1664).

### Caregiving status

The primary exposure was dichotomized by the informal caregiving status of the participants, according to the question, “Are you currently providing care to a person living together or to any relative not living with you?” As we used the panel data, when a participant who had answered “no (yes)” changed the answer to “yes (no)” in subsequent waves, we defined it as a “change in informal caregiving status.”

For those who responded that they were providing care, the survey asked them to fill out the average number of weekly hours they provided informal care. The secondary exposure was the average weekly hours of informal caregiving, grouped into three groups: 1–9 hours/week, 10–19 hours/week, and ≥20 hours/week, following the categorization used in prior research.^6^

### Outcomes

The outcomes were four types of self-reported health-related behaviors that may increase the risk of cardiovascular events: heavy drinking,^27^ smoking,^28^ no exercise habits,^29^ and non-attendance at annual health checkups.^30^ To account for the temporal relationship in which caregiving may change lifestyle, these outcomes were measured in the year following the exposure measurement year. Heavy drinking was dichotomized, defined as consumption of more than three *go* (540 mL) of Japanese sake (approximately equal to 60 g of ethanol) or the equivalent amount of alcohol per day with the halved threshold for women, based on previous studies.^31^^,^^32^ Smoking was dichotomized based on the answer to the question, “Do you currently smoke?” Moderate-to-intense exercise was a binary variable (once a month or more, no) based on the question about the frequency (twice or more per week, once a month to once a week, none) of “moderately energetic” (eg, walking, jogging) or “highly energetic” (eg, aerobics, swimming) exercise. Attendance at health checkups was dichotomized according to the question about the completion of health checkups during the previous year.

### Adjustment variables

We adjusted for time-variant and time-invariant characteristics of the participants. Time-varying characteristics were measured in the same year as the exposure measurement year. They included age (continuous) and categorical variables for marital status (married, not married), job status (self-employment, regular employee, non-regular employee, not working), household income level (high, middle, low, unanswered), comorbidities (diabetes, heart disease, stroke, hypertension, dyslipidemia, and cancer), and social capital (bonding and bridging).^33^ We included social capital as adjustment variables since social capital is reportedly associated with healthier behaviors^34^; moreover, social capital could alleviate a caregiver’s burden or intensity of care. To define the household income level, we calculated the monthly household equivalent income. We grouped it into four groups by the tertiles (high: more than 260,000 JPY per month, middle: 145,000 to 260,000 JPY per month, low: less than 145,000 JPY per month) and a category indicating respondents who did not answer about their income. When calculating the household equivalent income, we divided the sum of the monthly income of a respondent and their spouse by the square root of the number of family members living together. Bonding and bridging social capitals are both binary variables, based on the questions about their participation in social activities (hobbies, sports, community events, childcare support, educational or cultural activities, support for older people, and others) and about whom they do these social activities with. Based on a previous report,^33^ when the respondents answered that they joined social activities with family members, friends, colleagues, or neighbors, we considered them to have bonding social capital. If they answered that they do social activities with non-profit organizations or public interest cooperation, we considered them to have bridging social capital. Time-invariant characteristics included gender and educational attainment (junior high school, high school, and college or higher).

### Statistical analyses

To examine whether informal caregiving status was associated with health-related behaviors adjusting for potential confounders, we applied a multivariable logistic regression model with correlated random effects.^35^ We adjusted for the participants’ time-variant characteristics and participant-level averages of these time-variant variables (correlated random effects). This approach allowed us to eliminate confounding in the association between caregiving and outcomes owing to observed and unobserved individual characteristics. It is known that the correlation random effects approach produces the same estimates as the fixed effects approach, which is more commonly used to adjust for time-invariant variables when using linear regression to adjust for unobserved individual-level characteristics in a balanced panel data.^36^ However, when using non-linear regression, such as logistic regression, it has been reported that the correlated random effects approach is less biased than the fixed effects model (eg, conditional fixed effect logit) especially when there is a serial correlation (see eMaterial 1 for more details on the statistical analyses).^37^ For each outcome, we reported adjusted odds ratios (aORs) of informal caregiving status, which indicate the association between the change in informal caregiving and the change in each health-related behavior. We also calculated an adjusted probability for each behavior by using marginal standardization.^38^ We used the robust standard errors corrected for clustering at the individual level.

We conducted stratified analyses by gender, educational attainments (junior high school, high school, and college or higher), and the household income level (low, middle, and high) at baseline.

As our study had four types of outcomes, we calculated the adjusted *P*-values using the Benjamini-Hochberg method to reduce type 1 errors, with a statistical significance level of adjusted *P* < 0.05.^39^ We conducted all the analyses using Stata version 17 (StataCorp, College Station, TX, USA).

### Sensitivity analyses

We conducted several sensitivity analyses. First, we repeated the analyses excluding those with health-related behaviors at baseline (wave 1). Second, as the changes in caregiving status could affect lifestyle sooner than one year, we repeated the analyses by measuring the outcomes in the same year as the exposure measurement year instead of measuring in the year following the exposure measurement year. Third, although the main analysis (correlated random effects model) estimated the effect of the change in informal caregiving within the same individual, it assumed that the effects of “transition into” and “out of” caregiving had the same (symmetric) magnitude. To allow for the possibility that “transition into” and “out of” caregiving had asymmetric effects, we applied an asymmetric fixed effects model.^40^ Lastly, we evaluated the association between caregiving and health-related behaviors by the duration of caregiving (ie, years since the beginning of caregiving; <1 year or ≥1 year).

## RESULTS

### Characteristics of the participants

After excluding the 3,975 individuals with missing values or no participation in the two consecutive waves, we analyzed 30,530 individuals with 268,165 observations. The median age at baseline was 55 (the interquartile range, 52–57) years, and 51.6% were women (Table 1). We observed 10,224 individuals (33.5%) providing informal care in at least any one wave during the study period; caregivers were more likely to be women compared to non-caregivers. In wave 1, the prevalence of informal caregiving, heavy drinking, smoking, no exercise habits, and no attendance at health checkups was 8.5%, 5.9%, 29.1%, 65.2%, and 25.2%, respectively.

**Table 1.** Basic characteristics of the study participants, by informal caregiving status

**Time-variant variables**	Non-caregivers	Caregivers	Total
*N* (observations over 2005–2019)	236,001	32,164	268,165
Hours of informal caregiving per week			
1–9	—	15,649 (48.7)	—
10–19	—	5,668 (17.6)	—
≥20	—	8,910 (27.7)	—
Unanswered	—	1,937 (6.0)	—
Marital status, married	201,772 (85.5)	27,814 (86.5)	229,586 (85.6)
Job status			
Self-employment	35,230 (14.9)	4,861 (15.1)	40,091 (15.0)
Regular employee	61,611 (26.1)	6,336 (19.7)	67,947 (25.3)
Non-regular employee	63,826 (27.0)	7,797 (24.2)	71,623 (26.7)
Not working	75,334 (31.9)	13,170 (41.0)	88,504 (33.0)
Comorbidities			
Cancer	6,843 (2.9)	992 (3.1)	7,835 (2.9)
Cerebrovascular diseases	4,806 (2.0)	602 (1.9)	5,408 (2.0)
Diabetes	25,169 (10.7)	3,255 (10.1)	28,424 (10.6)
Heart diseases	11,228 (4.8)	1,530 (4.8)	12,758 (4.8)
Dyslipidemia	39,168 (16.6)	6,608 (20.5)	45,776 (17.1)
Hypertension	70,376 (29.8)	9,465 (29.4)	79,841 (29.8)
Self-rated health			
Good	193,880 (82.2)	25,153 (78.2)	219,033 (81.7)
Poor	42,121 (17.9)	7,011 (21.8)	49,132 (18.3)
Heavy drinking	11,838 (5.0)	1,516 (4.7)	13,354 (5.0)
Current smoking	47,665 (20.2)	5,190 (16.1)	52,855 (19.7)
Exercise habits			
No	140,085 (59.4)	19,021 (59.1)	159,106 (59.3)
Once a month–once a week	31,696 (13.4)	4,468 (13.9)	36,164 (13.5)
More than once a week	64,220 (27.2)	8,675 (27.0)	72,895 (27.2)
Attendance at annual health checkups	164,936 (69.9)	22,111 (68.7)	187,047 (69.8)
Monthly household equivalent income, thousand yen, median (IQR)	20.9 (13–31.5)	18.7 (11.3–29.3)	20.5 (12.7–31.2)
Bonding social capital	167,590 (71.0)	23,981 (74.6)	191,571 (71.4)
Bridging social capital	16,911 (7.2)	2,861 (8.9)	19,772 (7.4)

**Time-invariant variables**			

*N* (individuals)	20,306	10,224	30,530
Age in 2005, years, median (IQR)	55 (52–57)	55 (52–57)	55 (52–57)
Women	9,867 (48.6)	5,877 (57.5)	15,744 (51.6)
Educational Attainment			
Junior high school	4,177 (20.6)	1,297 (12.7)	5,474 (17.9)
High school, Junior college	13,075 (64.4)	7,032 (68.8)	20,107 (65.9)
College or higher	3,054 (15.0)	1,895 (18.5)	4,949 (16.2)

IQR, interquartile range.

Values reported as number (%) unless otherwise specified. Median monthly household equivalent income was calculated for 223,803 observations (26,398 caregivers and 197,405 non-caregivers) without missing values.

### Informal caregiving and health-related behaviors

After adjusting for time-variant confounders and individual-level time-invariant characteristics (Table 2), informal caregiving was associated with higher probabilities of health-related behaviors that may increase the risk of cardiovascular events, including heavy drinking (adjusted probability 5.2% vs 4.9%; aOR 1.16; 95% confidence interval [CI], 1.03–1.32; adjusted *P* = 0.032) and no exercise habits (adjusted probability 60.8% vs 59.6%; aOR 1.09; 95% CI, 1.04–1.15; adjusted *P* < 0.001). We observed similar patterns for smoking (adjusted probability 18.6% vs 18.3%; aOR 1.12; 95% CI, 1.001–1.26; adjusted *P* = 0.053) and no attendance at health checkups (adjusted probability 31.5% vs 30.9%; aOR 1.05; 95% CI, 0.999–1.10; adjusted *P* = 0.053), though the associations were not statistically significant.

**Table 2.** Association between informal caregiving and health-related behaviors

*N* = 268,165 observations (30,530 individuals)	Heavy drinking		Smoking		No exercise habits		No attendance at health checkups	

Independent variables	Adjusted OR (95% CI)	Adjusted probability, % (95% CI)	Adjusted OR (95% CI)	Adjusted probability, % (95% CI)	Adjusted OR (95% CI)	Adjusted probability, % (95% CI)	Adjusted OR (95% CI)	Adjusted probability, % (95% CI)
Informal caregiving, yes	1.16^*^ (1.03–1.32)	5.2 (4.8–5.6)	1.12 (1.001–1.26)	18.6 (17.6–19.6)	1.09^*^ (1.04–1.15)	60.8 (60.1–61.5)	1.05 (0.999–1.10)	31.5 (30.9–32.1)
Informal caregiving, no	reference	4.9 (4.6–5.2)	reference	18.3 (17.4–19.2)	reference	59.6 (59.2–60.0)	reference	30.9 (30.6–31.2)
Hours spent on informal caregiving (ref: no)								
1–9 hours/week	1.23^*^ (1.06–1.43)	5.3 (4.9–5.8)	1.02 (0.89–1.17)	18.4 (17.4–19.4)	1.05 (0.99–1.11)	60.2 (59.4–61.0)	1.01 (0.95–1.08)	31.0 (30.2–31.8)
10–19 hours/week	1.01 (0.79–1.29)	4.9 (4.3–5.5)	1.13 (0.93–1.36)	18.7 (17.6–19.7)	1.09 (0.998–1.19)	60.7 (59.5–61.9)	1.04 (0.95–1.14)	31.4 (30.3–32.5)
≥20 hours/week	1.09 (0.88–1.33)	5.1 (4.5–5.6)	1.41^*^ (1.15–1.74)	19.3 (18.2–20.4)	1.19^*^ (1.10–1.29)	61.9 (60.8–63.0)	1.12^*^ (1.04–1.22)	32.3 (31.3–33.4)
Informal caregiving, no	reference	4.9 (4.6–5.2)	reference	18.4 (17.4–19.3)	reference	59.6 (59.2–59.9)	reference	30.9 (30.5–31.2)

CI, confidence interval; OR, odds ratio.

^*^Adjusted *P* < 0.05. Caregiving status was dichotomized in model 1 and classified according to caregiving intensity (hours spent on caregiving) in model 2. In the models using the hours of informal caregiving per week as an outcome, we included 266,228 observations (30,493 individuals) that answered about their caregiving hours. We applied the correlated random effects model, adjusted for age, gender, educational attainment, marital status, job status, social capital, household income, comorbidities, and self-rated health. We used the Benjamini-Hochberg method to account for multiple comparisons (four outcomes).

When we subdivided caregiving status by the intensity of care, long hours of informal caregiving were primarily associated with health-related behaviors that undermine health. The associations with smoking (adjusted probability 19.3% vs 18.4%; aOR 1.41; 95% CI, 1.15–1.74; adjusted *P* = 0.006), no exercise habits (adjusted probability 61.9% vs 59.6%; aOR 1.19; 95% CI, 1.10–1.29; adjusted *P* < 0.001), and no attendance at annual health checkups (adjusted probability 32.3% vs 30.9%; aOR 1.12; 95% CI, 1.04–1.22; adjusted *P* = 0.020) were salient for caregiving of ≥20 hours per week compared to non-caregiving. However, the association with heavy drinking was found only among caregivers who spent 1–9 hours per week (adjusted probability 5.3% vs 4.9%; aOR 1.23; 95% CI, 1.06–1.43; adjusted *P* = 0.021).

### Informal caregiving and health-related behaviors by gender

**Figure 1.**
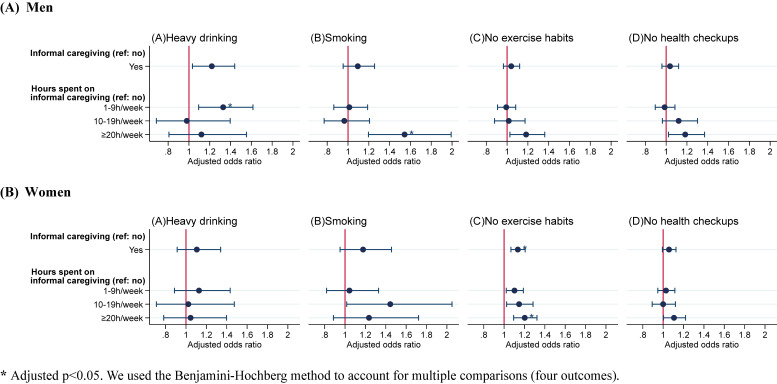
Association between informal caregiving and health-related behaviors by gender

Stratified analysis showed that the association between informal caregiving and health-related behaviors varied by gender (Figure 1). The association with no exercise habits was observed only for women providing care for ≥20 hours per week (aOR 1.20; 95% CI, 1.09–1.32; adjusted *P* < 0.001), while the associations of caregiving with heavy drinking and smoking were observed only among men. Men providing care for 1–9 hours per week had a higher odds ratio for heavy drinking (aOR 1.33; 95% CI, 1.09–1.62; adjusted *P* = 0.024), and those for ≥20 hours per week had an increased odds ratio of smoking (aOR 1.54; 95% CI, 1.20–1.99; adjusted *P* = 0.012). The adjusted odds ratios and confidence intervals for each model are presented in eTable 1.

### Informal caregiving and health-related behaviors by educational attainment

**Figure 2.**
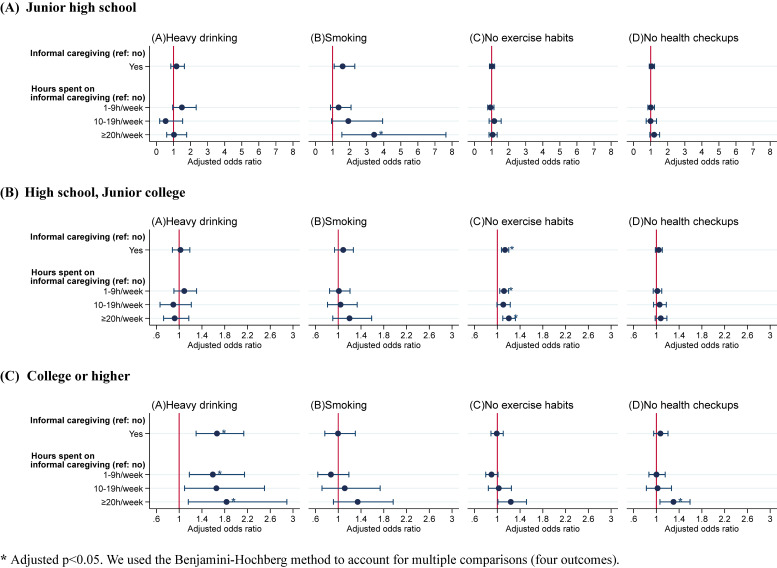
Association between informal caregiving and health-related behaviors by educational attainment

The types of health-related behaviors associated with the change in informal caregiving status were not consistent across educational attainment (Figure 2). For example, an increased odds ratio of informal caregiving for ≥20 hours per week for smoking was found only among junior high school graduates (aOR 3.44; 95% CI, 1.54–7.64; adjusted *P* = 0.024) while that for no exercise habits was found among high school graduates (aOR 1.20; 95% CI, 1.10–1.32; adjusted *P* < 0.001). The association of informal caregiving for ≥20 hours per week with heavy drinking (aOR 1.83; 95% CI, 1.16–2.89; adjusted *P* = 0.044) and no attendance at health checkups (aOR 1.30; 95% CI, 1.06–1.59; adjusted *P* = 0.044) were observed only among college graduates or higher. The adjusted odds ratios and confidence intervals for each model are presented in eTable 2.

### Informal caregiving and health-related behaviors by income level

Stratified by the household equivalent income at baseline (Table 3), we observed the association between informal caregiving and worsening of health-related behaviors in the middle- or high-income groups. In the middle-income group providing care for ≥20 hours per week, we found the higher odds ratio for smoking (aOR 1.88; 95% CI, 1.24–2.83; adjusted *P* = 0.03) and no exercise habits (aOR 1.22; 95% CI, 1.06–1.40; adjusted *P* = 0.03). In the highest income group, we found the increased odds ratio for heavy drinking (aOR 1.34; 95% CI, 1.09–1.65; adjusted *P* = 0.024) and no exercise habits (aOR 1.10; 95% CI, 1.01–1.19; adjusted *P* = 0.046) among caregivers.

**Table 3.** Association between informal caregiving and health-related behaviors, by household equivalent income at baseline

**(A) Low**								

*N* = 72,564 observations (9,132 individuals)	Heavy drinking		Smoking		No exercise habits		No attendance at health checkups	

Independent variables	Adjusted OR	95% CI	Adjusted OR	95% CI	Adjusted OR	95% CI	Adjusted OR	95% CI
Informal caregiving, yes (ref: no)	1.19	(0.95–1.48)	1.02	(0.80–1.31)	1.10	(0.9998–1.20)	1.06	(0.97–1.16)
Hours spent on informal caregiving (ref: no)								
1–9 hours/week	1.21	(0.92–1.59)	0.88	(0.07–10.7)	1.07	(0.95–1.21)	1.00	(0.89–1.12)
10–19 hours/week	1.30	(0.83–2.05)	0.81	(0.0004–1531.9)	1.05	(0.89–1.23)	1.09	(0.93–1.28)
≥20 hours/week	1.05	(0.76–1.44)	1.53	(0.0001–22775.9)	1.20	(1.04–1.38)	1.13	(0.99–1.30)

**(B) Middle**								

*N* = 86,677 observations (9,518 individuals)	Heavy drinking		Smoking		No exercise habits		No attendance at health checkups	

Independent variables	Adjusted OR	95% CI	Adjusted OR	95% CI	Adjusted OR	95% CI	Adjusted OR	95% CI

Informal caregiving, yes (ref: no)	0.997	(0.80–1.24)	1.23	(1.01–1.50)	1.09	(1.003–1.18)	1.05	(0.96–1.14)
Hours spent on informal caregiving (ref: no)								
1–9 hours/week	1.18	(0.90–1.55)	1.04	(0.83–1.31)	1.02	(0.93–1.13)	1.07	(0.96–1.19)
10–19 hours/week	0.58	(0.37–0.92)	1.28	(0.93–1.76)	1.09	(0.94–1.28)	0.99	(0.84–1.16)
≥20 hours/week	0.91	(0.62–1.32)	1.88^*^	(1.24–2.83)	1.22^*^	(1.06–1.40)	1.06	(0.91–1.22)

**(C) High**								

*N* = 93,474 observations (9,664 individuals)	Heavy drinking		Smoking		No exercise habits		No attendance at health checkups	

Independent variables	Adjusted OR	95% CI	Adjusted OR	95% CI	Adjusted OR	95% CI	Adjusted OR	95% CI

Informal caregiving, yes (ref: no)	1.34^*^	(1.09–1.65)	1.04	(0.82–1.30)	1.10^*^	(1.01–1.19)	1.04	(0.96–1.14)
Hours spent on informal caregiving (ref: no)								
1–9 hours/week	1.30	(1.02–1.66)	1.01	(0.78–1.30)	1.06	(0.97–1.17)	0.99	(0.89–1.10)
10–19 hours/week	1.25	(0.84–1.86)	1.18	(0.82–1.70)	1.12	(0.97–1.30)	1.02	(0.87–1.19)
≥20 hours/week	1.42	(0.97–2.09)	1.04	(0.72–1.52)	1.16	(1.01–1.34)	1.19	(1.03–1.39)

CI, confidence interval; OR, odds ratio.

^*^Adjusted *P* < 0.05. We categorized 28,314 individuals who answered the income at baseline into three groups, using the tertile of household equivalent income. In the models using the hours of informal caregiving per week as an outcome, we included 250,956 observations (28,281 individuals) that answered about their caregiving hours. We applied the correlated random effects model, adjusted for age, gender, educational attainment, marital status, job status, social capital, comorbidities, and self-rated health. We used the Benjamini-Hochberg method to account for multiple comparisons (four outcomes).

### Sensitivity analyses

First, when we limited the analyses to participants who did not have the negative health-related behaviors at baseline, we only observed the association between the informal caregiving and lack of exercise habits (aOR 1.12; 95% CI, 1.04–1.21), as provided in eTable 3. Second, our findings were largely unaffected by assessing the associations of informal caregiving with the health-related behaviors of the same wave (eTable 4, eTable 5, eTable 6, and eTable 7), except for no statistically significant associations with heavy drinking among college graduates or higher and no statistically significant association in the four outcomes across all the income groups. Third, the asymmetric effects model showed that the transition into caregiving was associated with a lack of exercise habits (aOR 1.09; 95% CI, 1.03–1.15). For the other three outcomes, the transition into caregiving had the point estimates of aORs >1, although they were mostly not statistically significant. The transition out of caregiving was not associated with any of the four outcomes (eTable 8). Fourth, by the duration of informal caregiving, caregiving started within a year was associated with smoking (aOR 1.16; 95% CI, 1.03–1.30), while prolonged caregiving for ≥1 year was associated with heavy drinking (aOR 1.27; 95% CI, 1.08–1.50) and no exercise habits (aOR 1.13; 95% CI, 1.06–1.20), as presented in eTable 9.

## DISCUSSION

Using the Japanese nationwide 15-year unbalanced panel data of 30,530 individuals, we found that change in informal caregiving status, especially transition into caregiving, was associated with adverse health-related behaviors. In particular, the caregivers with long hours (spending ≥20 hours per week) were more likely than non-caregivers to have smoking habits, less likely to have exercise habits, and less likely to attend health checkups. To the best of our knowledge, this is the first longitudinal study to investigate the association between the change in informal caregiving status—including its intensity—and a wide variety of health-related behaviors. From the public health perspective, our findings suggest the importance of ensuring informal caregivers the opportunity to receive their health checkups, particularly when providing care for long hours. Furthermore, health professionals involved in formal care services (eg, physicians, nurses, and care workers for home health care) should pay attention to changes in caregivers’ health-related behaviors and facilitate the use of respite care services.

There are several potential explanations as to why the transition into informal caregiving is associated with unhealthy lifestyles. First, informal caregivers—particularly when providing care for long hours—may have prioritized caregiving and could not afford to do exercise or attend checkups. Second, informal caregivers are reported to have higher levels of stress than non-caregivers,^41^ and informal caregivers may consume more alcohol and cigarettes to relieve stress related to caregiving. Regarding the intensity of caregiving, the association of informal caregiving with heavy drinking was observed only among the caregivers who spent 1–9 hours per week. The reason for the association with heavy drinking among caregivers with shorter hours was not apparent. One possibility is that caregivers for shorter hours may drink to cope with their stress. However, when they are required to provide care for longer hours, they may not continue to drink heavily because doing so could impair their concentration and disturb their caregiving ability.

With regard to the differences by caregiver’s gender, a prior Japanese study reported that men were more likely to cope with stress by drinking and smoking compared with women.^42^ Thus, the gender differences in stress-coping strategies may partly explain our findings of the increased heavy drinking and smoking only among men caregivers. Regarding educational attainment, the association between informal caregiving and health-related behaviors was inconsistent. In general, lower educational attainment is associated with unhealthy behaviors.^32^ However, our results suggest that adverse changes in caregivers’ health-related behaviors can occur, regardless of educational attainment. Stratified by the income level at baseline, we found deteriorating health-related behaviors associated with informal caregiving among the middle- and high-income groups but not among the low-income groups. This may seem counterintuitive, as it was previously reported that caregivers with lower incomes were more likely to experience depressive symptoms.^43^ One study also reported an increase in cardiovascular mortality among low-income caregivers.^44^ Our negative results for low-income caregivers may imply the presence of other mechanisms that can explain the increased cardiovascular diseases among low-income caregivers, other than the changes in health-related behaviors.

Finally, when we limited the analyses to those who did not have negative health-related behaviors at baseline, we observed the association of informal caregiving with a lack of exercise habits but not with other health-related behaviors. Individuals who did not have unhealthy lifestyle factors, such as smoking or heavy drinking habits, at baseline may not have started these behaviors even when commencing with informal caregiving.

Our study adds to a limited body of literature evaluating the association between informal caregiving and health-related behaviors. Thus far, several cross-sectional studies reported mixed results: some reported that a higher intensity of caregiving was associated with being overweight and smoking,^13^ and with lack of enough rest or exercise,^20^ while others found no differences in the use of preventive clinical services.^25^ These studies could not rule out the possibility of confounding by unobserved participants’ characteristics; for example, those who do not participate in the labor market because of poor health may be more likely to engage in informal care and also to have adverse health-related behaviors. In the current longitudinal study, we demonstrated the higher odds of heavy drinking, smoking, no exercise habits, and no attendance at health checkups among informal caregivers even after accounting for unobserved characteristics of the participants by comparing outcomes within the same participants. These changes in health-related behaviors may be one of the possible mechanisms for the reported increases in coronary heart diseases among long-hour informal caregivers.^6^^,^^7^

Most existing reports are from the United States and Europe. Therefore, the present study from Japan is noteworthy for several reasons. First, Japan introduced the long-term care insurance system in 2000 to relieve the burden of informal caregivers.^45^ Even so, the current study suggested that the transition into informal caregiving was associated with changes in health-related behaviors that could adversely affect their health. Second, we found several different results from foreign studies, particularly in the subgroup analyses. A previous longitudinal study of 17 European countries reported increased alcohol drinking, particularly among out-of-home caregivers with lower educational levels.^12^ In spite of this, we found increased odds for heavy drinking only among informal caregivers who graduated from college or higher. We were unable to identify the reasons for the inconsistencies; however, they may reflect cultural differences between Europe and Japan.

Our results suggested that informal caregiving, albeit moderate, is an independent factor associated with health-related behaviors that could increase the risk of cardiovascular diseases. These findings may aid in understanding the underlying mechanisms of health deterioration among informal caregivers and in considering policy interventions to alleviate their burden. Our study has some limitations. First, we did not have detailed information on the conditions of the care recipients, including their underlying diseases, activity of daily living, or the type of formal services that they used. Second, although we adjusted for time-varying observed characteristics as well as time-invariant observed or unobserved characteristics, there remains a possibility of residual confounding owing to unobserved time-varying variables. Nevertheless, the data containing a wide variety of variables regarding demographic and socioeconomic status allowed us to adjust for potential confounders when evaluating the association between informal caregiving and health-related behaviors. Furthermore, unlike most existing research with a cross-sectional design, this longitudinal study using a panel survey for 15 years was able to avoid the possibility of reverse causality. Third, we could not account for health-related behaviors or caregiving experiences before the study period. Fourth, as our study population was aged 50–59 years at baseline, we could not generalize the findings to other age groups. Fifth, the follow-up rate at the end of the 15th survey was 48.8%, and selection bias caused by the drop-out is possible. Sixth, the validity of some variables, such as income or caregiving hours, may be limited because they were self-reported. Seventh, although we evaluated the weekly hours of informal caregiving, we could not differentiate the frequency (eg, spending 1 hour daily vs 7 hours once a week). Finally, this study examined short-term changes in health-related behaviors owing to caregiving status; future studies on the long-term changes are warranted.

### Conclusion

Using a nationwide panel survey of Japan for 15 years, this study demonstrated that the transition into informal caregiving was associated with deterioration in the caregivers’ health-related behaviors. Our findings suggest the importance of attention to caregivers’ health-related behaviors, which could adversely affect their health.

## SUPPLEMENTARY MATERIAL

eMaterial 1. Multivariable logistic regression model with correlated random effects.

eTable 1. Association between informal caregiving and health-related behaviors by gender.

eTable 2. Association between informal caregiving and health-related behaviors by educational attainment.

eTable 3. Association between informal caregiving and health-related behaviors among those who did not have health-related behaviors at baseline.

eTable 4. Association between informal caregiving and health-related behaviors (sensitivity analysis).

eTable 5. Association between informal caregiving and health-related behaviors, by gender (sensitivity analysis).

eTable 6. Association between informal caregiving and health-related behaviors, by educational attainment (sensitivity analysis).

eTable 7. Association between informal caregiving and health-related behaviors, by household equivalent income at baseline (sensitivity analysis).

eTable 8. Association between informal caregiving and health-related behaviors in the asymmetric fixed-effects model.

eTable 9. Association between informal caregiving and health-related behaviors, by the duration of caregiving.
