# Supplementary material for: Association Between Informal Caregiving and Changes in Cardiovascular-related Health Behaviors Among Middle-aged and Older Adults in Japan: A 15-year Panel Survey
Source: J Epidemiol. 2025 Aug 5;35(8):355–63. doi: 10.2188/jea.JE20240197 (PMC12237587; doi:10.2188/jea.JE20240197)
Supplement: Supplementary file 1 [file je-35-355-s001.pdf]

eMaterial 1. Multivariable logistic regression model with correlated random effects.

eTable 1. Association between informal caregiving and health-related behaviors by gender.

eTable 2. Association between informal caregiving and health-related behaviors by educational attainment.

eTable 3. Association between informal caregiving and health-related behaviors among those who did not have health-related behaviors at baseline.

eTable 4. Association between informal caregiving and health-related behaviors (sensitivity analysis).

eTable 5. Association between informal caregiving and health-related behaviors, by gender (sensitivity analysis).

eTable 6. Association between informal caregiving and health-related behaviors, by educational attainment (sensitivity analysis).

eTable 7. Association between informal caregiving and health-related behaviors, by household equivalent income at baseline (sensitivity analysis).

eTable 8. Association between informal caregiving and health-related behaviors in the asymmetric fixed-effects model.

eTable 9. Association between informal caregiving and health-related behaviors, by the duration of caregiving.

eMaterial 1. Multivariable logistic regression model with correlated random effects

To account for individual-level unobserved characteristics, we applied the multivariable logistic regression model with correlated random effects. In panel data analysis for binary outcome variables, previous research (Kwak et al., 2023) showed that using a correlated random effects approach to adjust for individual-level unobserved characteristics is less biased than a fixed effect model (e.g., conditional fixed effect logit), especially when serial correlation exists.

For the multivariable logistic regression analysis with correlated random effects, we estimated the following model:

$$log\left(\frac{p_{it+1}}{1-p_{it+1}}\right) = \mu_t + \beta x_{it} + \gamma \overline{x_i} + \delta z_i + \alpha_i \qquad (1)$$

where p_{it} denotes the probability of the i-th participant having the outcomes (i.e., health-related behaviors) in year t is equal to 1; x_{it} represents whether the i-th participant has each time-variant exposure (i.e., informal caregiving status and covariates) in year t and $\overline{x_i}$ indicates the mean of x_i; z_i represents the time-invariant variables of the i-th participant (i.e, gender and educational attainment); α_i stands for the combined effects of all unmeasured characteristics of the i-th participant that are constant over time; and μ_t is an intercept which could be different in each period.

By including participant-level averages of each covariate ($\overline{x_i}$) as adjustment variables of the regression models, we were able to effectively remove the effects of observed and unobserved time-invariant individual characteristics (Wooldridge, 2018).

REFERENCES

Kwak, D.W., Martin, R.S., Wooldridge, J.M., 2023. The Robustness of Conditional Logit for Binary Response Panel Data Models with Serial Correlation. Journal of Econometric Methods 12, 33–56.

Wooldridge, J.M., 2018. Introductory Econometrics: a Modern Approach.

eTable 1. Association between informal caregiving and health-related behaviors by gender

(A) Men

N=129,412 observations (14,786 individuals)	Heavy drinking		Smoking		No exercise habits		No attendance at health checkups	
Independent variables	Adjusted OR	95% CI	Adjusted OR	95% CI	Adjusted OR	95% CI	Adjusted OR	95% CI
Informal caregiving, yes (ref: no)	1.22	(1.03–1.44)	1.09	(0.95–1.26)	1.04	(0.96–1.12)	1.04	(0.96–1.12)
Hours spent on informal caregiving (ref: no)								
1–9 hours/week	1.33*	(1.09–1.62)	1.01	(0.86–1.19)	0.99	(0.91–1.08)	0.99	(0.90–1.08)
10–19 hours/week	0.98	(0.69–1.40)	0.96	(0.77–1.21)	1.02	(0.88–1.17)	1.12	(0.96–1.30)
≥20 hours/week	1.12	(0.81–1.55)	1.54*	(1.20–1.99)	1.18	(1.03–1.36)	1.18	(1.02–1.37)

(B) Women

N=138,753 observations (15,744 individuals)	Heavy drinking		Smoking		No exercise habits		No attendance at health checkups	
Independent variables	Adjusted OR	95% CI	Adjusted OR	95% CI	Adjusted OR	95% CI	Adjusted OR	95% CI
Informal caregiving, yes (ref: no)	1.11	(0.91–1.34)	1.18	(0.95–1.46)	1.13*	(1.07–1.21)	1.06	(0.99–1.13)
Hours spent on informal caregiving (ref: no)								
1–9 hours/week	1.13	(0.89–1.43)	1.04	(0.82–1.33)	1.10	(1.02–1.19)	1.03	(0.95–1,11)
10–19 hours/week	1.02	(0.71–1.47)	1.44	(1.02–2.05)	1.15	(1.02–1.28)	1.00	(0.89–1.12)
≥20 hours/week	1.05	(0.78–1.40)	1.24	(0.89–1.72)	1.20*	(1.09–1.32)	1.11	(1.003–1.22)

CI, confidence interval; OR, odds ratio.

*Adjusted $p<0.05$. Caregiving status was dichotomized in model 1 and classified according to caregiving intensity (hours spent on caregiving) in model 2. In the models using the hours of informal caregiving per week as an outcome, we included 266,228 observations (128,619 for men and 137,609 for women) that answered about their caregiving hours. We applied the correlated random effects model, adjusted for age, educational attainment, marital status, job status, social capital, household income, comorbidities, and self-rated health. We used the Benjamini-Hochberg method to account for multiple comparisons (four outcomes).

eTable 2. Association between informal caregiving and health-related behaviors by educational attainment

(A) Junior high school

N=38,396 observations (5,474 individuals)	Heavy drinking		Smoking		No exercise habits		No attendance at health checkups	
Independent variables	Adjusted OR	95% CI	Adjusted OR	95% CI	Adjusted OR	95% CI	Adjusted OR	95% CI
Informal caregiving, yes (ref: no)	1.17	(0.84–1.64)	1.59	(1.10–2.30)	1.01	(0.87–1.17)	1.06	(0.92–1.22)
Hours spent on informal caregiving (ref: no)								
1–9 hours/week	1.49	(0.95–2.33)	1.35	(0.88–2.09)	0.94	(0.77–1.14)	1.01	(0.83–1.22)
10–19 hours/week	0.54	(0.19–1.54)	1.92	(0.94–3.93)	1.15	(0.86–1.56)	0.99	(0.74–1.34)
≥20 hours/week	1.03	(0.60–1.77)	3.44*	(1.54–7.64)	1.06	(0.85–1.33)	1.20	(0.95–1.52)

(B) High school, Junior college

N=181,211 observations (20,107 individuals)	Heavy drinking		Smoking		No exercise habits		No attendance at health checkups	
Independent variables	Adjusted OR	95% CI	Adjusted OR	95% CI	Adjusted OR	95% CI	Adjusted OR	95% CI
Informal caregiving, yes (ref: no)	1.02	(0.88–1.19)	1.09	(0.93–1.27)	1.14*	(1.07–1.20)	1.04	(0.98–1.10)
Hours spent on informal caregiving (ref: no)								
1–9 hours/week	1.09	(0.91–1.31)	1.01	(0.85–1.21)	1.12*	(1.04–1.20)	1.02	(0.94–1.10)
10–19 hours/week	0.90	(0.66–1.21)	1.04	(0.81–1.33)	1.11	(0.99–1.23)	1.06	(0.95–1.18)
≥20 hours/week	0.92	(0.73–1.17)	1.20	(0.90–1.59)	1.20*	(1.10–1.32)	1.08	(0.98–1.19)

(C) College or higher

N=48,558 observations (4,949 individuals)	Heavy drinking		Smoking		No exercise habits		No attendance at health checkups	
Independent variables	Adjusted OR	95% CI	Adjusted OR	95% CI	Adjusted OR	95% CI	Adjusted OR	95% CI
Informal caregiving, yes (ref: no)	1.66*	(1.30–2.13)	0.996	(0.76–1.30)	0.99	(0.89–1.10)	1.07	(0.95–1.20)
Hours spent on informal caregiving (ref: no)								
1–9 hours/week	1.59*	(1.18–2.15)	0.87	(0.64–1.19)	0.90	(0.80–1.01)	1.001	(0.87–1.15)
10–19 hours/week	1.65	(1.09–2.50)	1.11	(0.71–1.74)	1.03	(0.84–1.25)	1.02	(0.82–1.27)
≥20 hours/week	1.83*	(1.16–2.89)	1.34	(0.91–1.97)	1.24	(1.01–1.51)	1.30*	(1.06–1.59)

CI, confidence interval; OR, odds ratio.

*Adjusted $p<0.05$. Caregiving status was dichotomized in model 1 and classified according to caregiving intensity (hours spent on caregiving) in model 2. In the models using the hours of informal caregiving per week as an outcome, we included 266,228 observations that answered about their caregiving hours. We applied the correlated random effects model, adjusted for age, gender, marital status, job status, social capital, household income, comorbidities, and self-rated health. We used the Benjamini-Hochberg method to account for multiple comparisons (four outcomes).

eTable 3. Association between informal caregiving and health-related behaviors among those who did not have health-related behaviors at baseline

	Heavy drinking N=221,557 observations (23,942 individuals)		Smoking N=170,880 observations (17,972 individuals)		No exercise habits N=80,412 observations (8,227 individuals)		No attendance at health checkups N=180,512 observations (19,021 individuals)	
Independent variables	Adjusted OR	(95% CI)	Adjusted OR	(95% CI)	Adjusted OR	(95% CI)	Adjusted OR	(95% CI)
Informal caregiving, yes (ref: no)	1.12	(0.96–1.30)	1.09	(0.67–1.77)	1.12*	(1.04–1.21)	1.05	(0.98–1.11)
Hours spent on informal caregiving (ref: no)								
1–9 hours/week	1.16	(0.96–1.41)	0.76	(0.48–1.21)	1.12	(1.02–1.23)	1.02	(0.94–1.10)
10–19 hours/week	0.96	(0.70–1.32)	1.21	(0.63–2.30)	1.07	(0.92–1.24)	1.02	(0.91–1.14)
≥20 hours/week	1.13	(0.87–1.45)	1.82	(0.81–4.08)	1.26*	(1.10–1.44)	1.12	(1.01–1.25)

CI, confidence interval; OR, odds ratio.

*Adjusted $p<0.05$. Caregiving status was dichotomized in model 1 and classified according to caregiving intensity (hours spent on caregiving) in model 2. We applied the correlated random effects model, adjusted for age, gender, educational attainment, marital status, job status, social capital, household income, comorbidities, and self-rated health. We used the Benjamini-Hochberg method to account for multiple comparisons (four outcomes).

eTable 4. Association between informal caregiving and health-related behaviors (sensitivity analysis)

	Heavy drinking		Smoking		No exercise habits		No attendance of health checkups	
Independent variables	Adjusted OR	95% CI	Adjusted OR	95% CI	Adjusted OR	95% CI	Adjusted OR	95% CI
Informal caregiving, yes (ref: no)	1.11	(0.97–1.28)	1.17*	(1.04–1.33)	1.09*	(1.03–1.16)	1.09*	(1.02–1.15)
Hours spent on informal caregiving (ref: no)								
1–9 hours/week	1.10	(0.93–1.29)	1.13	(0.96–1.33)	1.02	(0.95–1.09)	1.03	(0.95–1.11)
10–19 hours/week	1.06	(0.81–1.39)	1.10	(0.87–1.40)	1.13	(1.02–1.25)	1.12	(1.01–1.25)
≥20 hours/week	1.10	(0.86–1.40)	1.28	(0.99–1.65)	1.23*	(1.11–1.35)	1.18*	(1.07–1.30)

CI, confidence interval; OR, odds ratio.

*Adjusted $p<0.05$. We applied the correlated random effects model, adjusted for age, gender, educational attainment, marital status, job status, social capital, household income, comorbidities, and self-rated health. We used the Benjamini-Hochberg method to account for multiple comparisons (four outcomes).

eTable 5. Association between informal caregiving and health-related behaviors, by gender (sensitivity analysis)

(A) Men

	Heavy drinking		Smoking		No exercise habits		No attendance of health checkups	
Independent variables	Adjusted OR	95% CI	Adjusted OR	95% CI	Adjusted OR	95% CI	Adjusted OR	95% CI
Informal caregiving, yes (ref: no)	1.08	(0.90–1.31)	1.10	(0.95–1.26)	0.98	(0.90–1.07)	1.09	(0.99–1.20)
Informal caregiving, no	Reference		Reference		Reference		Reference	
Hours spent on informal caregiving (ref: no)								
1–9 hours/week	1.45*	(1.08–1.95)	1.04	(0.89–1.22)	0.91	(0.82–1.02)	1.01	(0.90–1.14)
10–19 hours/week	1.33*	(1.07–1.67)	1.01	(0.80–1.26)	1.07	(0.90–1.27)	1.13	(0.94–1.35)
≥20 hours/week	1.45*	(1.17–1.81)	1.34	(1.04–1.74)	1.12	(0.94–1.33)	1.30*	(1.10–1.54)

(B) Women

	Heavy drinking		Smoking		No exercise habits		No attendance of health checkups	
Independent variables	Adjusted OR	95% CI	Adjusted OR	95% CI	Adjusted OR	95% CI	Adjusted OR	95% CI
Informal caregiving, yes (ref: no)	1.16	(0.76–1.79)	1.30*	(1.04–1.63)	1.18*	(1.09–1.27)	1.08	(1.004–1.17)
Hours spent on informal caregiving (ref: no)								
1–9 hours/week	1.18	(0.79–1.76)	1.33	(0.87–2.01)	1.11	(1.01–1.22)	1.03	(0.94–1,14)
10–19 hours/week	1.06	(0.69–1.64)	1.26	(0.71–2.25)	1.18	(1.03–1.34)	1.12	(0.98–1.28)
≥20 hours/week	1.21	(0.44–3.35)	1.23	(0.84–1.80)	1.28*	(1.13–1.44)	1.14	(1.01–1.29)

CI, confidence interval; OR, odds ratio.

*Adjusted $p<0.05$. We applied the correlated random effects model, adjusted for age, educational attainment, marital status, job status, social capital, household income, comorbidities, and self-rated health. We used the Benjamini-Hochberg method to account for multiple comparisons (four outcomes).

eTable 6. Association between informal caregiving and health-related behaviors, by educational attainment (sensitivity analysis)

(A) Junior high school

	Heavy drinking		Smoking		No exercise habits		No attendance of health checkups	
Independent variables	Adjusted OR	95% CI	Adjusted OR	95% CI	Adjusted OR	95% CI	Adjusted OR	95% CI
Informal caregiving, yes (ref: no)	1.07	(0.73–1.57)	1.63*	(1.14–2.33)	1.09	(0.90–1.33)	1.13	(0.95–1.34)
Hours spent on informal caregiving (ref: no)								
1–9 hours/week	0.92	(0.54–1.58)	1.35	(0.97–1.87)	0.96	(0.75–1.24)	1.23	(0.97–1.54)
10–19 hours/week	0.43	(0.17–1.07)	1.07	(0.56–2.03)	1.25	(0.87–1.79)	1.16	(0.84–1.61)
≥20 hours/week	1.11	(0.59–2.12)	3.63*	(1.75–7.54)	1.23	(0.90–1.67)	1.04	(0.78–1.38)

(B) High school, Junior college

	Heavy drinking		Smoking		No exercise habits		No attendance of health checkups	
Independent variables	Adjusted OR	95% CI	Adjusted OR	95% CI	Adjusted OR	95% CI	Adjusted OR	95% CI
Informal caregiving, yes (ref: no)	1.06	(0.89–1.26)	1.15	(0.96–1.38)	1.10*	(1.03–1.17)	1.06	(0.99–1.14)
Hours spent on informal caregiving (ref: no)								
1–9 hours/week	1.11	(0.91–1.36)	1.17	(0.94–1.44)	1.04	(0.96–1.13)	1.02	(0.93–1.11)
10–19 hours/week	1.04	(0.74–1.45)	1.27	(0.95–1.71)	1.05	(0.93–1.19)	1.07	(0.95–1.22)
≥20 hours/week	1.01	(0.74–1.36)	1.06	(0.77–1.45)	1.22*	(1.09–1.37)	1.15	(1.03–1.29)

(C) College or higher

	Heavy drinking		Smoking		No exercise habits		No attendance of health checkups	
Independent variables	Adjusted OR	95% CI	Adjusted OR	95% CI	Adjusted OR	95% CI	Adjusted OR	95% CI
Informal caregiving, yes (ref: no)	1.31	(0.97–1.79)	0.98	(0.73–1.33)	1.07	(0.94–1.22)	1.15	(0.999–1.32)
Hours spent on informal caregiving (ref: no)								
1–9 hours/week	1.14	(0.80–1.62)	0.91	(0.64–1.29)	0.96	(0.83–1.11)	0.97	(0.81–1.15)
10–19 hours/week	1.45	(0.88–2.40)	0.82	(0.51–1.33)	1.36	(1.09–1.70)	1.28	(0.99–1.67)
≥20 hours/week	1.45	(0.84–2.50)	1.22	(0.76–1.96)	1.22	(0.97–1.55)	1.48*	(1.16–1.90)

CI, confidence interval; OR, odds ratio.

*Adjusted $p<0.05$. We applied the correlated random effects model, adjusted for age, gender, marital status, job status, social capital, household income, comorbidities, and self-rated health. We used the Benjamini-Hochberg method to account for multiple comparisons (four outcomes).

.

eTable 7. Association between informal caregiving and health-related behaviors, by household equivalent income at baseline (sensitivity analysis)

(A) Low

	Heavy drinking		Smoking		No exercise habits		No attendance of health checkups	
Independent variables	Adjusted OR	95% CI	Adjusted OR	95% CI	Adjusted OR	95% CI	Adjusted OR	95% CI
Informal caregiving, yes (ref: no)	1.20	(0.94–1.53)	1.34	(1.05–1.70)	1.09	(0.98–1.22)	1.12	(1.01–1.25)
Hours spent on informal caregiving (ref: no)								
1–9 hours/week	1.14	(0.83–1.55)	1.27	(0.95–1.70)	1.00	(0.87–1.16)	1.10	(0.96–1.26)
10–19 hours/week	1.22	(0.78–1.91)	1.47	(1.02–2.11)	1.11	(0.92–1.35)	1.11	(0.92–1.34)
≥20 hours/week	1.16	(0.77–1.73)	1.28	(0.91–1.79)	1.19	(0.999–1.42)	1.17	(0.99–1.38)

(B) Middle

	Heavy drinking		Smoking		No exercise habits		No attendance of health checkups	
Independent variables	Adjusted OR	95% CI	Adjusted OR	95% CI	Adjusted OR	95% CI	Adjusted OR	95% CI
Informal caregiving, yes (ref: no)	0.95	(0.73–1.22)	1.12	(0.90–1.39)	1.13*	(1.03–1.25)	1.09	(0.98–1.20)
Hours spent on informal caregiving (ref: no)								
1–9 hours/week	0.92	(0.68–1.23)	1.09	(0.87–1.37)	1.05	(0.94–1.18)	1.08	(0.95–1.23)
10–19 hours/week	0.72	(0.46–1.14)	1.17	(0.85–1.60)	1.08	(0.89–1.31)	1.08	(0.90–1.30)
≥20 hours/week	1.13	(0.69–1.85)	1.17	(0.76–1.81)	1.30*	(1.10–1.54)	1.16	(0.97–1.39)

(C) High

	Heavy drinking		Smoking		No exercise habits		No attendance of health checkups	
Independent variables	Adjusted OR	95% CI	Adjusted OR	95% CI	Adjusted OR	95% CI	Adjusted OR	95% CI
Informal caregiving, yes (ref: no)	1.19	(0.94–1.52)	0.97	(0.77–1.23)	1.04	(0.94–1.14)	1.10	(0.98–1.22)
Hours spent on informal caregiving (ref: no)								
1–9 hours/week	1.25	(0.94–1.65)	0.95	(0.70–1.29)	0.98	(0.87–1.09)	0.97	(0.85–1.11)
10–19 hours/week	1.27	(0.79–2.05)	0.78	(0.45–1.34)	1.17	(0.99–1.39)	1.21	(0.997–1.46)
≥20 hours/week	0.98	(0.66–1.46)	1.26	(0.73–2.17)	1.18	(0.99–1.41)	1.26	(1.05–1.51)

CI, confidence interval; OR, odds ratio.

*Adjusted p<0.05. We applied the correlated random effects model, adjusted for age, gender, educational attainment, marital status, job status, social capital, comorbidities, and self-rated health. We used the Benjamini-Hochberg method to account for multiple comparisons (four outcomes).

eTable 8. Association between informal caregiving and health-related behaviors in the asymmetric fixed-effects model (sensitivity analysis)

	Transition into informal caregiving		Transition out of informal caregiving	
	Adjusted OR	95% CI	Adjusted OR	95% CI
Heavy drinking	1.14	(0.98–1.33)	0.97	(0.84–1.13)
Smoking	1.14	(0.94–1.39)	1.14	(0.94–1.37)
No exercise habits	1.09*	(1.03–1.15)	0.94	(0.89–0.998)
No attendance at health checkups	1.02	(0.96–1.08)	0.96	(0.90–1.02)

CI, confidence interval; OR, odds ratio.

*Adjusted $p<0.05$. We applied the asymmetric fixed-effects model, adjusting for age, gender, educational attainment, marital status, job status, social capital, household income, comorbidities, and self-rated health.

eTable 9. Association between informal caregiving and health-related behaviors, by the duration of caregiving

N=268,165 observations (30,530 individuals)	Heavy drinking		Smoking		No exercise habits		No attendance at health checkups	
Independent variables	Adjusted OR	95% CI	Adjusted OR	95% CI	Adjusted OR	95% CI	Adjusted OR	95% CI
Years since the beginning of informal caregiving (ref: no)								
<1 year	1.08	(0.94–1.24)	1.16*	(1.03–1.30)	1.06	(1.01–1.12)	1.03	(0.98–1.09)
≥1 year	1.27*	(1.08–1.50)	1.07	(0.90–1.27)	1.13*	(1.06–1.20)	1.07	(1.002–1.14)

CI, confidence interval; OR, odds ratio.

*Adjusted p<0.05. We applied the correlated random effects model, adjusted for age, gender, educational attainment, marital status, job status, social capital, household income, comorbidities, and self-rated health. When a respondent who had answered" not providing care" changed the answer to "providing care" in the next wave, we defined it as caregiving <1 year. When a respondent answered "providing care" for two or more consecutive waves, we defined it as caregiving ≥1 year.
